# Mushroom Volatile Compounds Mediate Avoidance Behavior in a Mycophagous Slug and Differential Responses in *Drosophila* Flies

**DOI:** 10.3390/ani16101444

**Published:** 2026-05-08

**Authors:** Keiko Kitabayashi, Yuri Nishiwaki-Akine, Nobuko Tuno

**Affiliations:** 1 Natural Science and Technology, Kanazawa University, Kanazawa 920-1192, Japan; 2 Transdisciplinary Sciences for Innovation, Kanazawa University, Kanazawa 920-1192, Japan

**Keywords:** mushroom volatile compounds, chemical ecology, slug, fungal–animal interaction, *Amanita*

## Abstract

Volatile organic compounds (VOCs) emitted by mushrooms may influence interactions with animal consumers, yet the roles of individual compounds and their mixtures remain poorly understood. This study examined odor-mediated behavioral responses of a mycophagous slug and three drosophilid species to mushroom-derived VOCs. Slug feeding avoidances were tested using 43 mushroom species, and tolerance to α-amanitin was evaluated to assess whether avoidance of toxic mushrooms was related to toxin sensitivity. Headspace volatiles from 22 mushroom species (48 samples) were analyzed using Gas Chromatography-Mass spectrometry (GC/MS), and behavioral assays were conducted to test responses of slugs and flies to selected VOCs and their mixtures. The slug showed complete avoidance only of *Amanita pallidorosea* and *Russula subnigricans*, and all individuals survived within the tested α-amanitin dose range, indicating that avoidance was not explained by toxin sensitivity. GC/MS detected 65 VOCs, and fragmented *Amanita* samples formed a distinct group characterized by sulfide-containing compounds. Dimethyl trisulfide induced strong avoidance in slugs, and repellency increased when compounds were combined. Among flies, avoidance responses were most pronounced in *Drosophila melanogaster*. These findings indicate that mushroom VOCs can function as chemical repellents and that behavioral sensitivity varies among consumers.

## Simple Summary

Mushrooms release a variety of smells, which are often thought to attract animals that feed on them, but these odors may also serve to repel some consumers. In this study, we investigated how a fungus-feeding slug and three species of *Drosophila* flies respond to mushroom odors. Feeding tests using 43 mushroom species showed that the slug completely avoided only two species. This avoidance was not explained by sensitivity to toxins, as the slugs survived exposure to amanitin, a toxic substance produced by some mushrooms. We then analyzed the smells released by 22 mushroom species and identified many different odor compounds. Mushrooms that were damaged or decaying produced sulfur-containing compounds, which are typically associated with strong, unpleasant smells. Behavioral experiments showed that one of these compounds caused strong avoidance in slugs, especially at higher concentrations. Odor mixtures from intact mushrooms also increased the repellent effect. Among the flies, species that do not normally feed on fungi showed clear avoidance responses, whereas species that commonly feed on fungi did not. These results suggest that mushroom odors can act as chemical signals that deter certain animals, and that different species vary in how they respond to these cues.

## 1. Introduction

Fungal volatile organic compounds (VOCs) are widespread chemical cues that mediate interactions between fungi and animals [1,2,3]. Produced during growth and sporulation, these compounds include alcohols, ketones, aldehydes, and terpenoids that disperse through the environment [1,2,4]. Although fungal VOCs have been widely studied in plant–fungus and microbe–microbe systems, their ecological roles in animals remain comparatively less understood.

Macroscopic fungal fruiting bodies (mushrooms) emit complex odor blends that vary among species and developmental stages. Some mushrooms attract animals that disperse spores, whereas others appear to deter consumers. Stinkhorn fungi (Phallaceae), for example, release dimethyl oligosulfides that attract flies responsible for spore dispersal [5]. Comparative analyses of mushroom volatiles reveal substantial interspecific variation in both shared and unique components [6].

Mycophagous gastropods rely heavily on fungi as food and use chemical cues detected by sensory structures on their tentacles and body surface. Although gastropod chemoreceptors remain poorly characterized at the molecular level, behavioral and electrophysiological studies demonstrate high sensitivity to characteristic fungal C8 compounds such as 1-octen-3-ol and 3-octanone [7]. These compounds often function as cues guiding feeding and orientation [8]. *Meghimatium fruhstorferi* is known to be mycophagous [9], yet detailed information on its mushroom avoidances remains limited. To identify fungal species or ecological groups that attract or repel this slug, we conducted feeding avoidance experiments.

In *Drosophila*, olfactory perception is mediated by odorant receptors expressed in antennal neurons, several of which respond to fungal-associated volatiles including 1-octen-3-ol [10]. Behavioral responses, however, are context dependent. For example, 1-octen-3-ol can act as an attractant in some ecological contexts but may induce avoidance at higher concentrations or in other taxa [10,11]. These responses reflect concentration-dependent processing and neural integration rather than a fixed behavioral meaning [12,13,14].

We investigated the effects of mushroom odors on slugs and, for comparison, also examined their effects on drosophilid flies. Although slugs and drosophilid flies are phylogenetically distant, both are major consumers of mushrooms in temperate forests in Japan [15,16] and have also been reported as such in North America [17]. If mushroom odors function in communication with animals, examining the responses of both slugs and drosophilid flies may help elucidate these interspecific interactions.

Volatile compounds may be avoided either because they are inherently harmful or because they signal the presence of harmful substances. Many mushrooms are known to be toxic, particularly those in the genus *Amanita* [18,19]. Based on our field observations, flies frequently aggregated on mushrooms of the genus *Amanita* [19,20], whereas feeding damage by slugs was rarely observed. Therefore, in this study, we quantified slug avoidance of mushrooms and, at the same time, evaluated their sensitivity to α-amanitin, a toxin commonly found in *Amanita* species and known to be highly toxic to eukaryotes [19,20].

In this study, we investigated odor-mediated interactions between mushrooms and animals using a mycophagous slug (*Meghimatium fruhstorferi*) and three drosophilid flies (*Drosophila melanogaster*, *D. angularis*, and *D. busckii*). By combining feeding assays, chemical analyses of mushroom VOCs, and behavioral bioassays, we aimed to identify odor compounds associated with attraction or avoidance and to determine whether responses differ among animal consumers. Volatile collection from fruiting bodies was conducted under two sample conditions, using intact and fragmented fruiting bodies, and in both cases headspace volatiles were collected. In both conditions, immature and mature fruiting bodies were analyzed when available. Fragmented samples were used to collect volatiles associated with tissue damage and decay.

## 2. Materials and Methods

###  2.1. Avoidance of *Meghimatium fruhstorferi* for Mushroom Species

Field collections were carried out in June–July 2016 and 2017 in a forest dominated by *Quercus variabilis* and *Quercus serrata* on the Kanazawa University campus in Ishikawa Prf., west central Japan. Slugs and mushroom fruiting bodies were collected simultaneously. Slugs were identified by a specialist, and mushrooms were identified using standard field guides [21,22]. *Amanita pallidorosea* was confirmed by DNA analysis.

Fruiting bodies were categorized by developmental stage as follows: immature (cap unopened), mature (cap expanded and actively dispersing spores), and senescent (cap recurved and discolored). Mushrooms were classified ecologically as either ectomycorrhizal fungi, which form mutualistic associations with plant roots and obtain carbon from their hosts, or saprotrophs (wood-decaying and litter-decomposing fungi), which derive nutrients by breaking down dead organic matter.

Collected slugs were maintained individually in plastic cups containing moist sphagnum moss at 22 °C under long-day conditions (14 h light:10 h dark) and fed carrots and cucumbers. Prior to experiments, slugs were starved for three days with access to water only.

Avoidance assays were conducted at 25 °C in plastic trays (25 × 35 × 5 cm). One mushroom fruiting body and five starved slugs were introduced into each tray, with slugs initially placed 15 cm from the mushroom. Although using specimens of identical size would be ideal, this was not feasible because wild mushrooms were used. Therefore, specimens of similar size were selected as much as possible. Only undamaged mushrooms (e.g., without feeding marks) were used at the start of the experiments. Fresh weight ranged from approximately 20 to 70 g. Feeding behavior was observed visually and recorded using interval photography (WG-20, Ricoh Co., Ltd., Tokyo, Japan) at 1 min intervals for 15 min. The avoidance rate was defined as:

$$\mathrm{Avoidance}\;\mathrm{rate}\;\left(\%\right)=\frac{\left(\mathrm{Number}\;\mathrm{of}\;\mathrm{individuals}\;\mathrm{that}\;\mathrm{did}\;\mathrm{not}\;\mathrm{feed}\right)\times 100}{\mathrm{Total}\;\mathrm{number}\;\mathrm{of}\;\mathrm{individuals}}$$

The number of slugs and fungal species tested are shown in Table 1.

### 2.2. Evaluation of α-Amanitin Tolerance in *Meghimatium fruhstorferi*

Based on our field observations, flies frequently aggregated on mushrooms of the genus *Amanita*, whereas feeding damage by slugs was rarely observed. Therefore, in this study, we quantified slug avoidance of mushrooms and, at the same time, evaluated their sensitivity to α-amanitin, a toxin commonly found in *Amanita* species and known to be highly toxic to eukaryotes. α-Amanitin, a toxin produced by some species of *Amanita* species, i.e., *Amanita phalloides*, inhibits mRNA synthesis required for protein production [23]. The LD_50_ in humans is 0.1 mg/kg [24]. Because amanitin tolerance has not been investigated in slugs, we evaluated its toxicity in *M. fruhstorferi*. Aqueous α-amanitin solutions (C_39_H_54_N_10_O_14_S; MW 918.97; Fujifilm Wako, Osaka, Japan) were prepared at concentrations of 1000 µg/µL, 100 µg/µL, 10 µg/µL, and 0 µg/µL as control. Each slug received 20 µL of sterile water solution (N = 4, 4, 3, and 5 per concentration) into the dorsal body cavity at a 30° angle using a 31G needle [25] and monitored the survival daily for one week.

### 2.3. Analysis of Mushroom Volatile Compounds

To compare volatile profiles components between *Amanita pallidorosea* and other mushroom species and to select candidate repellent compounds, volatile analyses of wild mushrooms were conducted. Fruiting bodies were collected from forests in Ishikawa Prefecture, Japan, from July to October between 2016 and 2018.

Fragmented samples were used to collect volatiles associated with tissue damage and decay. A 3 g (fresh weight) piece of the mushroom cap was placed in a sterilized glass bottle (MT Extract Cup, GL Sciences Inc., Tokyo, Japan). A carbon adsorbent chip (MonoTrap DCC18, GL Sciences Inc., Tokyo, Japan) was placed inside the bottle, which was then kept at 22 °C for 24–72 h. Intact samples were used to collect volatiles emitted from undamaged fruiting bodies under natural conditions, including stages at which spore dispersal was possible. An entire undamaged fruiting body was placed in a sterilized 500 mL glass bottle together with a MonoTrap DCC18 carbon chip (GL Sciences, Tokyo, Japan) and kept at 22 °C for 24 h.

After volatile collection by either method, the carbon chip was immersed in 500 µL of acetone and subjected to ultrasonic extraction for 10 min. The acetone extract containing the volatiles was transferred entirely to a 1.5 mL GC/MS vial (Shimadzu Co., Ltd., Kyoto, Japan) and subjected to gas chromatography–mass spectrometry (GC/MS) analysis.

GC/MS conditions were as follows: GCMS-QP2010 SE (Shimadzu Co., Ltd.); column: DB-5MS, 30 m × 0.25 mm i.d. × 0.25 µm (Agilent Technologies, Santa Clara, CA, USA). The oven temperature was held at 40 °C for 5 min, then increased from 40 °C to 250 °C at 4 °C/min, and held at 250 °C for 5 min. Injector temperature was 250 °C; injection mode was split (split ratio 10:1); carrier gas was helium at a flow rate of 1.62 mL/min. Mass spectra were obtained using electron ionization (EI) at 70 eV, with a scan range of *m*/*z* 25–450.

Compounds were identified by comparing mass spectra with those in the NIST11 library attached to the GC/MS system. In addition, Kovats retention indices were calculated using retention times of a standard mixture of n-paraffins (TCI; C7–C11, C12–C16, C17–C20), and compound identities were further confirmed by comparison with values in the NIST database. Hierarchical cluster analysis of volatile composition among fungal species, maturation stages, and collection methods was conducted using JMP ver. 14.1 (SAS Institute Inc., Cary, NC, USA). To identify compounds potentially responsible for avoidance behavior in *Meghimatium fruhstorferi*, candidate compounds were selected from VOCs detected in *Amanita pallidorosea*, the mushroom species that showed complete avoidance in the feeding assay.

### 2.4. Behavioral Responses of *Meghimatium fruhstorferi* to Volatile Compounds

Bioassays were conducted using candidate repellent compounds selected from these VOCs. From fragmented samples, 1-pentanol, phenylethyl alcohol, and dimethyl trisulfide were selected. From intact fruiting bodies, 1-pentanol, 3-octanone, 2-propyl-1-pentanol, and ethyl butyrate were selected. Experiments were performed at 23 ± 1 °C in the laboratory. A transparent vinyl tube (3 cm in diameter, 15 cm in length) was placed inside a clean plastic container (25 × 35 × 5 cm). A single *M. fruhstorferi* individual was introduced at one end of the tube, with its head oriented toward the interior of the tube. Based on our observations, individuals move in the direction their head is facing. When the slug reached the midpoint of the tube (7.5 cm), a piece of absorbent cotton impregnated with 500 µL of a volatile test solution was placed at the distal end of the tube in the direction of movement. If the slug exited the tube from the end containing the test compound, the response was recorded as “no repellent effect.” If the slug reversed direction within the tube (turning approximately 180°) and returned to the original introduction end, the response was recorded as “repellent effect.” Observations were terminated 10 min after placement of the volatile solution. Individuals who remained within the tube without reaching either end were excluded from the analysis. Ten individuals were tested for each compound.

Each test solution consisted of either a single odor compound or an equal-volume mixture of multiple candidate compounds, diluted in liquid paraffin to a concentration of 1 µL/mL. To evaluate the effect of each odorant, logistic regression analysis was conducted with avoidance behavior as the response variable and presence/absence of the odor compound as the explanatory variable (JMP ver. 14.1, SAS Institute Inc., Cary, NC, USA). For dimethyl trisulfide, which showed a significant repellent effect, additional bioassays were conducted at four concentrations: 1 µL/mL, 0.1 µL/mL, 0.01 µL/mL and 0.001 µL/mL.

### 2.5. Behavioral Responses of Three *Drosophila* Species to Volatile Compounds

Using the same candidate compounds that induced avoidance behavior in the slug, bioassays were conducted on three *Drosophila* species with different feeding habits. Flies were obtained from KYORIN-Fly *Drosophila* Species Stock Center and reared at 23 ± 1 °C. The next generation of the obtained individuals was used for experiments.

The three species tested were: the fruit (yeast)-feeding *Drosophila melanogaster* (CS strain, Kyorin-fly), the decaying plant (bacteria)-feeding *D. busckii* (MIT1), and the mushroom (filamentous fungi)-feeding *D. angularis* (FK05-14) [26].

Bioassays were conducted at 23 ± 1 °C using transparent plastic tubes (2 cm in diameter and 15 cm in length). Ten or 20 active flies were introduced into each tube using an aspirator. Cotton plugs were placed at both ends of the tube. One end was treated with 500 µL of the test solution, diluted to 1 µL/mL in colorless, odorless liquid paraffin, and the other end was treated with 500 µL of liquid paraffin as a control. The tube was placed horizontally and left undisturbed for 5 min, after which the number of flies on the test-compound side and the control side, relative to the midpoint (7.5 cm), was recorded. The tube was then gently shaken to redistribute the flies, and counts were repeated after a further 5 min. This procedure was conducted three times within each trial using the same group of flies. The repeated counts were used to calculate a single Attractive Score for each trial. When 20 active individuals were available, 20 flies were used to obtain a more stable estimate of behavioral response. The number of individuals on the test and control sides was used to calculate the Attractive Score as follows:

$$\mathrm{Attractive}\;\mathrm{Score}=\frac{\left(\mathrm{Number}\;\mathrm{on}\;\mathrm{test}\;\mathrm{side}-\mathrm{Number}\;\mathrm{on}\;\mathrm{control}\;\mathrm{side}\right)}{\mathrm{Total}\;\mathrm{number}\;\mathrm{of}\;\mathrm{individuals}}$$

Attractive Scores for the three fly species were compared among odorants using Bonferroni-corrected multiple comparisons. For each fly species, responses to odorants were compared with the control using chi-square tests. In addition, to account for differences in sample size among trials (10 or 20 individuals), pooled raw count data were reanalyzed using Fisher’s exact test. Statistical analyses were performed using R version 3.4.3 [27].

Ethical review and approval were waived for this study because the experiments involved invertebrates that are not subject to institutional animal care regulations in Japan.

## 3. Results

### 3.1. Mushroom Avoidances

Avoidance was tested using fruiting bodies from 43 species (7 orders, 15 families, 23 genera). The overall mean avoidance rate was 36.15 ± 4.30% (mean ± SE) (Table 1).

Complete avoidance (100%) was observed only for immature (*n* = 15) and mature (*n* = 25) *Amanita pallidorosea* and mature *Russula subnigricans* (*n* = 10). In all cases, slugs approached the fruiting bodies, extended their tentacles at close range, and then retreated without feeding.

No saprotrophic fungal species were completely avoided. Although relatively high avoidance rates were recorded for *Inonotus mikadoi* (80%) and immature *Trametes versicolor* (60%), individuals that contacted these mushrooms began feeding without showing avoidance behavior, suggesting that some slugs may not have detected the fruiting bodies, rather than avoidance within the 15 min observation period. Comparison between immature and mature stages within species revealed a marginal difference only for *Trametes versicolor* (χ^2^ test, *p* = 0.068). No significant differences were found in four *Amanita* species (*A. pallidorosea*, *A. clarisquamosa*, *A. spissacea*, *A. fulva*) (χ^2^ test, *p* > 0.6).

### 3.2. α-Amanitin Tolerance

All injected individuals survived for one week at all concentrations tested α-Amanitin solution (1000 µg/µL, 100 µg/µL, 10 µg/µL, and water control). Assuming a slug’s average body weight of 10 g, the human LD_50_ (0.1 mg/kg) corresponds to 1 µg for a slug of equivalent mass. However, slugs survived injections of 20 µL of 1000 µg/µL solution (20,000 µg total), indicating tolerance exceeding 20,000-fold relative to human LD_50_ on a body-weight basis [24].

### 3.3. Mushroom Volatile Compounds

GC/MS analyses were conducted on 48 samples (Table S1) representing fruiting bodies of 22 species belonging to 11 genera, 8 families, and 4 orders, classified by species, maturation stage, and collection method. A total of 65 volatile compounds were detected (Table S2-1, S2-2). Compounds detected in more than half of the samples included 1-pentanol (26 samples), 2-methyl-1-butanol (22 samples), 3-octanone (17 samples), and 1,2-dichlorobenzene (14 samples).

**Figure 1:**
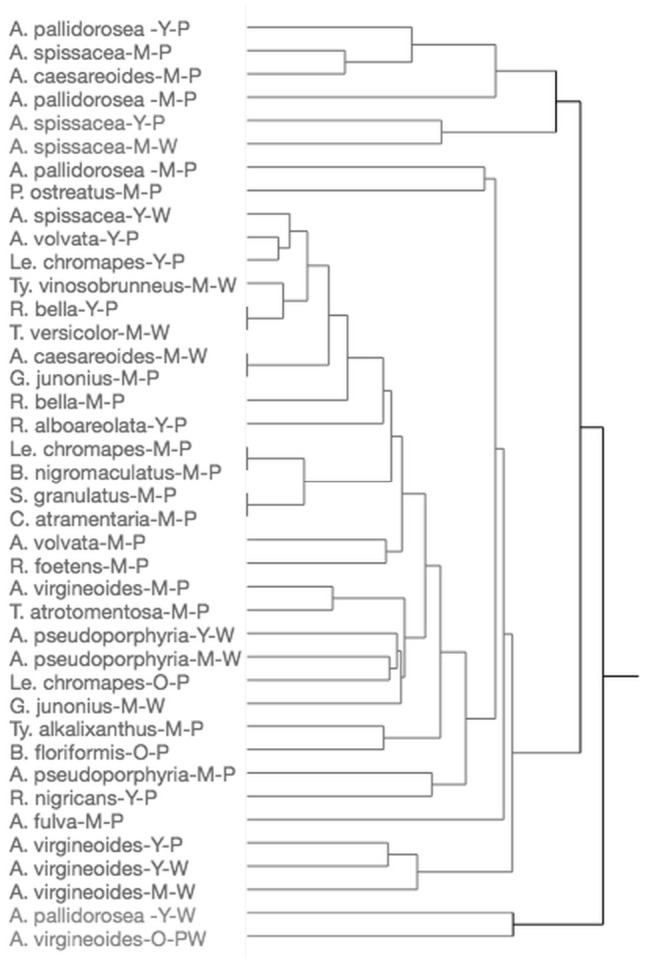
Cluster analysis of volatile compound profiles by mushroom species, maturation stage, and collection method.

To determine whether patterns existed in volatile composition among mushrooms, hierarchical cluster analysis was conducted based on volatile profiles according to species, maturation stage, and collection method. As a result, *Amanita pallidorosea* (immature–fragment), *A. pallidorosea* (mature–fragment), *Amanita spissacea* (mature–fragment), and *Amanita caesareoides* (mature–fragment) formed a cluster (Figure 1). Within this cluster, which included fragmented samples of immature and mature *A. pallidorosea*, mature *A. caesareoides*, and immature and mature *A. spissacea*, sulfide-containing compounds typically associated with decay odors were detected from all fragment samples.

To identify compounds responsible for the 100% avoidance rate shown by slugs toward fragmented *A. pallidorosea*, three compounds detected from these samples were selected as candidate repellents: 1-pentanol, phenylethyl alcohol, and dimethyl trisulfide (Table S3). 1-Pentanol is emitted by many fungi, including non-mushroom-forming molds such as *Aspergillus* and *Penicillium* [28]. Phenylethyl alcohol, a component of rose fragrance, was selected because its odor resembled the characteristic scent of *A. pallidorosea*. Dimethyl trisulfide (DMTS) has a strong pungent odor and is also present in truffles (*Tuber* spp., Tuberaceae) and stinkhorns (*Phallus* spp., Phallaceae) [29]. Among the mushrooms examined, DMTS was detected in *A. pallidorosea* and two additional species.

From intact fruiting bodies of *A. pallidorosea* during the spore-maturation stage, four compounds were selected as candidate repellents: 1-pentanol, 3-octanone, 2-propyl-1-pentanol, and ethyl butyrate (Table S4). Like 1-pentanol, 3-octanone is also emitted by molds such as *Penicillium* and *Aspergillus* [30,31]. In this study, 2-propyl-1-pentanol and ethyl butyrate were detected exclusively from *A. pallidorosea.* Ethyl butyrate is known as a fruity odor commonly used as a food flavoring agent.

### 3.4. Responses of *Meghimatium fruhstorferi* to Volatile Compounds

Bioassays were conducted using candidate repellent compounds derived from fragment samples, representing odors associated with damaged tissue and decay. Because avoidance might result from combinations of compounds, both single compounds and mixtures were tested.

**Figure 2:**
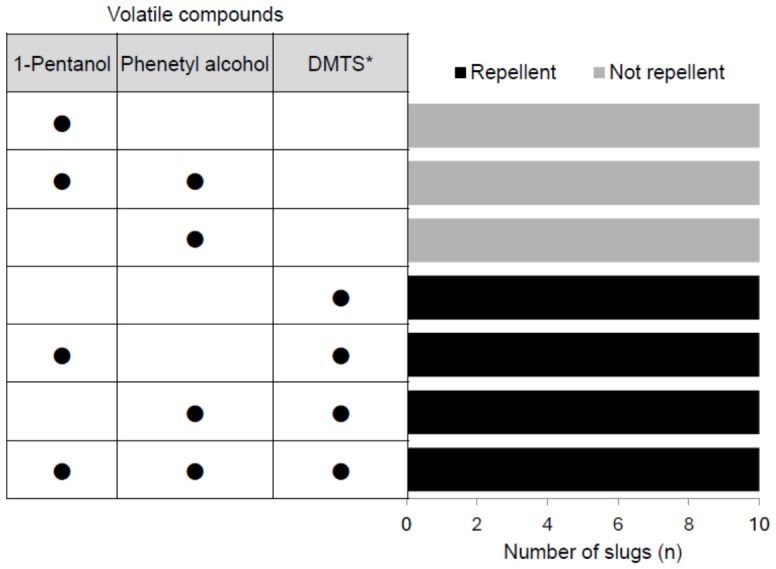
Behavioral responses of slugs to candidate repellent compounds selected from fragments of *Amanita pallidorosea*. DMTS*: Dimethyl trisulfide.

The slug exhibited a 100% avoidance rate (*n* = 40) toward dimethyl trisulfide alone and toward mixtures containing DMTS (Figure 2). In contrast, single compounds or mixtures that did not contain DMTS elicited 0% avoidance. Behavioral observations revealed that all *M. fruhstorferi* individuals approached the cotton containing DMTS, stopped at close range, repeatedly extended and retracted their tentacles, and then reversed direction.

**Figure 3:**
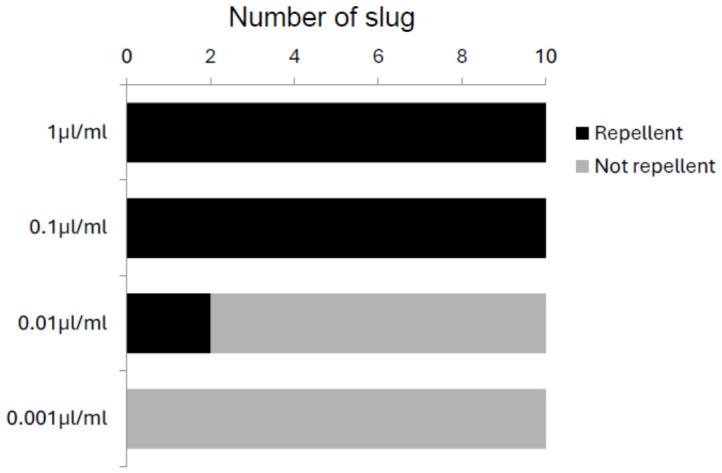
Avoidance responses of slugs (*n* = 10) to different concentrations of dimethyl trisulfide (DMTS).

To examine whether the avoidance response depended on DMTS concentration, slugs were tested with concentrations ranging from 0.001 µL/mL to 1 µL/mL in 10-fold increments. Avoidance rates were 100% at 0.1 µL/mL and 1 µL/mL but decreased to 20% at 0.01 µL/mL and 0% at 0.001 µL/mL (Figure 3), indicating a concentration-dependent response.

**Figure 4:**
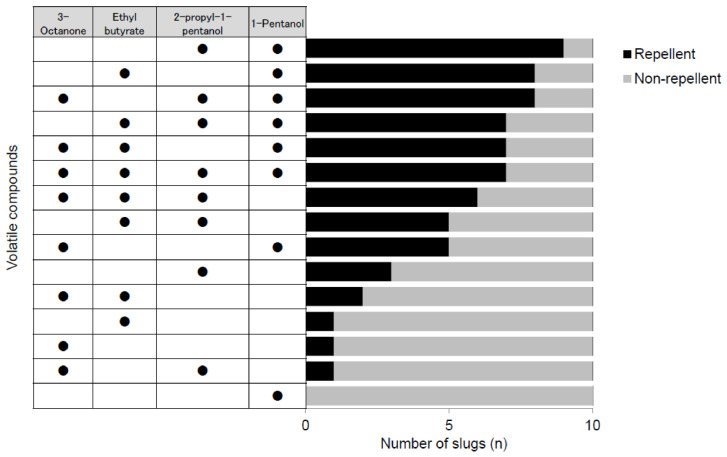
Responses of slugs to candidate repellent volatile compounds from intact fruiting bodies of *Amanita pallidorosea*.

Bioassays using candidate compounds detected from intact fruiting bodies (spore-dispersal stage) showed relatively low avoidance rates (≤30%) for single compounds. However, avoidance tended to increase when compounds were combined. For example, 1-pentanol alone elicited 0% avoidance, whereas a blend of 1-pentanol and 2-propyl-1-pentanol elicited the highest avoidance rate (90%) (Figure 4).

Logistic regression analysis indicated significant repellent effects of 1-pentanol (*p* < 0.0001), ethyl butyrate (*p* = 0.0068), and 2-propyl-1-pentanol (*p* = 0.0023). Synergistic effects were observed for blends of 3-octanone + 2-propyl-1-pentanol (*p* = 0.031) and 3-octanone + ethyl butyrate + 2-propyl-1-pentanol (*p* = 0.0228) (Table 2).

### 3.5. Responses of Three *Drosophila* Species to Volatile Compounds

When compounds derived from fragment samples (decay-stage odors) were tested, neither *D. angularis* nor *D. busckii* showed significant differences from controls for any odor tested (chi-square test, *p* > 0.05) (Table S5). In contrast, *D. melanogaster* showed significantly higher avoidance rates toward blends containing 1-pentanol, specifically 1-pentanol + dimethyl trisulfide (*p* < 0.005), 1-pentanol + phenylethyl alcohol (*p* < 0.001), and 1-pentanol + dimethyl trisulfide + phenylethyl alcohol (*p* < 0.001).

**Figure 5:**
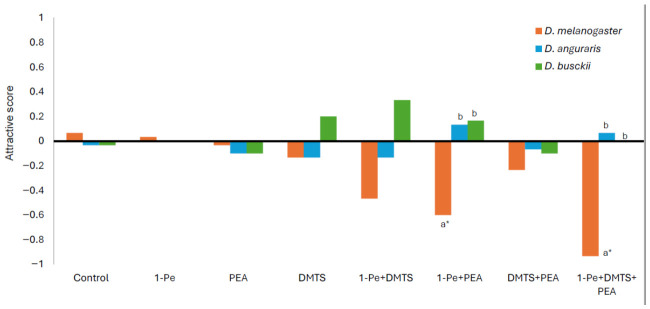
Behavioral responses of three *Drosophila* species to candidate repellent volatile compounds identified from fragments of *Amanita pallidorosea*. Attractive scores were calculated as (T − C)/(T + C), where T is the number of flies on the treatment side, and C is the number on the control side. Different letters indicate significant differences among species based on Bonferroni-corrected multiple comparisons (*p* < 0.05). Asterisks indicate significant differences from the control treatment. 1-Pe: 1-pentanol; PEA: phenylethyl alcohol. DMTS: dimethyl trisulfide.

Comparisons among the three fly species revealed significant differences between *D. melanogaster* and the other two species for the blends 1-pentanol + phenylethyl alcohol (Bonferroni test, *p* < 0.05) and 1-pentanol + phenylethyl alcohol + dimethyl trisulfide (*p* < 0.01) (Figure 5).

**Figure 6:**
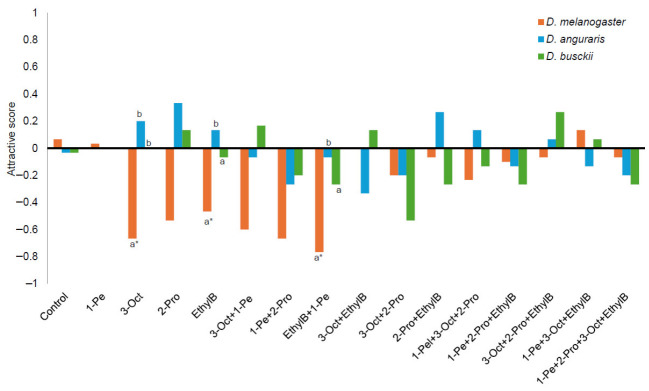
Behavioral responses of three *Drosophila* species to combinations of volatile compounds detected from intact fruiting bodies of *Amanita pallidorosea*. Attractive scores were calculated as (T − C)/(T + C), where T is the number of flies on the treatment side, and C is the number on the control side. Different letters indicate significant differences among species based on Bonferroni-corrected multiple comparisons (*p* < 0.05). Asterisks indicate significant differences from the control treatment. Abbreviations: 1-Pe, 1-pentanol; 3-Oct, 3-octanone; 2-Pro, 2-propyl-1-pentanol; EthylB, ethyl butyrate.

Similarly, when compounds derived from intact fruiting bodies (spore-dispersal stage) were tested, *D. angularis* and *D. busckii* showed no significant differences from controls (chi-square test, *p* > 0.05) (Table S6, Figure 6). In contrast, *D. melanogaster* exhibited significant avoidance responses to 3-octanone (*p* < 0.001), 2-propyl-1-pentanol (*p* < 0.001), ethyl butyrate (*p* < 0.001), 1-pentanol + 3-octanone (*p* < 0.001), 1-pentanol + 2-propyl-1-pentanol (*p* < 0.001), and 1-pentanol + ethyl butyrate (*p* < 0.001).

Comparisons of Attractive Scores among the three fly species showed that *D. melanogaster* differed significantly from the other two species in response to 3-octanone (Bonferroni test, *p* < 0.01). Significant differences between *D. melanogaster* and *D. angularis* were also observed for ethyl butyrate (*p* = 0.050) and ethyl butyrate + 1-pentanol (*p* = 0.013).

## 4. Discussion

This study demonstrates that volatile compounds emitted by mushrooms can function not only as attractants but also as chemical deterrents for animal consumers. In feeding assays, the mycophagous slug *M. fruhstorferi* completely avoided two poisonous mushroom species, *A. pallidorosea* and *R. subnigricans*. Behavioral experiments further showed that several mushroom-derived volatile compounds, particularly dimethyl trisulfide (DMTS), elicited strong avoidance responses. These findings indicate that mushroom VOCs may play an important role in mediating interactions between fungi and animal consumers.

Avoidance behavior toward *A. pallidorosea* cannot be explained by physiological sensitivity to its toxin α-amanitin. Slugs showed extremely high tolerance to injected α-amanitin, surviving doses far exceeding the lethal dose reported for humans. This result indicates that the observed avoidance behavior is more likely mediated by olfactory cues rather than direct toxic effects.

Avoidance assays indicated that *M. fruhstorferi* avoided the odor of *A. pallidorosea*. Behavioral experiments showed that volatile compounds emitted from intact fruiting bodies—including 1-pentanol, ethyl butyrate, and 2-propyl-1-pentanol—also produced repellent effects. Although the odor profile changed with mushroom maturation, the slug avoided feeding on this species at all developmental stages.

These results are consistent with the possibility that odor cues influence interactions between *A. pallidorosea* and animal consumers. However, the present data does not directly demonstrate adaptive ecological functions such as defense or optimization of spore dispersal. One plausible interpretation is that repellent odors reduce consumption by large consumers such as slugs, which could otherwise remove substantial portions of the hymenium. At the same time, alternative explanations should be considered, including the possibility that these odors reflect general metabolic or decay-related processes rather than evolved signaling functions. Previous studies have shown that dipteran larvae developing in ectomycorrhizal fruiting bodies can transport spores into soil, thereby facilitating mycorrhiza formation [32]. For ectomycorrhizal fungi, transport by dipteran larvae burrowing underground may represent a more effective dispersal pathway than consumption by large terrestrial gastropods.

During the decay stage, *A. pallidorosea* emitted DMTS, a compound known to attract saprophagous insects [33,34]. Time-lapse observations indicated that dung beetles visited decayed fruiting bodies. Such temporally dynamic odor emissions may influence the composition of visiting organisms [26,35,36,37]. However, whether these patterns reflect adaptive strategies or arise as by-products of decomposition processes requires further investigation.

Behavioral responses differed among the three *Drosophila* species examined. The frugivorous *D. melanogaster* showed stronger avoidance responses to several mushroom-derived compounds, including 1-pentanol and 3-octanone, whereas the more fungus-associated species *D. angularis* and *D. busckii* showed weaker responses. Such interspecific variation may reflect differences in ecological specialization, which could be shaped by evolutionary processes. Previous studies have shown that fungal contamination can negatively affect the survival and development of *D. melanogaster* larvae [38,39], suggesting that avoidance of certain fungal volatiles may be beneficial in this species. Compounds such as 1-pentanol, produced by filamentous fungi including *Aspergillus* and *Penicillium* [30,31], may therefore act as cues indicating unsuitable substrates for *D. melanogaster* but not for the other two saprophagous species.

## 5. Conclusions

This study demonstrates that volatile compounds emitted by mushrooms can function as chemical cues influencing the behavior of animal consumers. The mycophagous slug *Meghimatium fruhstorferi* showed strong avoidance of *Amanita pallidorosea*, and behavioral assays indicated that specific volatile compounds, particularly dimethyl trisulfide, contributed to this response. Additional compounds emitted from intact fruiting bodies also produced repellent effects, especially when present as odor blends. In contrast, behavioral responses of Drosophilid flies differed among species, with the strongest avoidance observed in frugivorous *D. melanogaster*. These findings suggest that mushroom volatile compounds may play multiple ecological roles, including defense against certain consumers and regulation of interactions with different animal species. Understanding such odor-mediated interactions may provide new insights into the ecological relationships between fungi and animals.

## Figures and Tables

**Table 1:** Avoidance rate (%) of slugs to fruiting bodies of 43 fungal species. Avoidance rates (%) are calculated as the proportion of slugs that did not feed within 15 min.

Order	Family	Genus	Species	Development Stages
Agaricales	Amanitaceae	*Amanita*	*Amanita pallidorosea*	mature
				immature
			*Amanita virosa*	mature
			*Amanita sychnopyramis*	mature
			*Amanita neoovoidea*	mature
			*Amanita pantherina*	mature
			*Amanita* sp.	mature
			*Amanita virgineoides*	immature
			*Amanita clarisquamosa*	mature
				immature
			*Amanita spissacea*	mature
				immature
			*Amanita pseudoporphyria*	mature
			*Amanita vaginata*	mature
			*Amanita caesareoides*	mature
			*Amanita longistriata*	mature
			*Amanita fulva*	mature
			*Amanita fulva*	immature
	Cortinariaceae	*Cortinarius*	*Cortinarius purpurascens*	mature
	Hydnangiaceae	*Laccaria*	*Laccaria vinaceoavellanea*	mature
	Physalacriaceae	*Armillaria*	*Armillaria tabescens*	mature
	Pleurotaceae	*Pleurotus*	*Pleurotus ostreatus*	mature
	Tricholomataceae	*Paralepistopsis*	*Paralepistopsis acromelalga*	mature
Boletales	Boletaceae	*Tylopilus*	*Tylopilus alkalixanthus*	immature
			*Tylopilus rigens*	mature
			*Tylopilus ferrugineus*	mature
			*Tylopilus castanoides*	mature
			*Tylopilus ballouii*	mature
		*Strobilomyces*	*Strobilomyces confusus*	mature
		*Boletellus*	*Boletellus floriformis*	mature
		*Leccinum*	*Leccinum chromapes*	mature
		*Boletus*	*Boletus subtomentosus*	mature
		*Xerocomus*	*Xerocomus parvulus*	mature
	Tapinellaceae	*Tapinella*	*Tapinella atrotomentosa*	mature
	Rhizopogonaceae	*Rhizopogon*	*Rhizopogon roseolus*	mature
Russulales	Russulaceae	*Russula*	*Russula subnigricans*	mature
			*Russula xerampelina*	mature
		*Lactarius*	*Lactarius glaucescens*	mature
			*Lactarius volemus*	mature
Cantharellales	Cantharellaceae	*Cantharellus*	*Cantharellus cibarius*	mature
Thelephorales	Thelephoraceae	*Thelephora*	*Thelephora aurantiotincta*	mature
Hymenochaetales	Hymenochaetaceae	*Inonotus*	*Inonotus mikadoi*	mature
Polyporales	Polyporaceae	*Trametes*	*Trametes versicolor*	mature
				immature
		*Microporus*	*Microporus vernicipes*	mature
		*Trametes*	*Trametes orientalis*	mature
	Hymenochaetaceae	*Trichaptum*	*Trichaptum biforme*	mature
	Podoscyphaceae	*Abortiporus*	*Abortiporus biennis*	mature

**Table 2:** Logistic regression results for avoidance responses of slugs to candidate repellent odorants selected from intact fruiting bodies of *Amanita pallidorosea*.

Odorant	No. of Parameters	df	*p* Value
1-Pentanol	1	1	<0.0001
3-Octanone	1	1	0.135
Ethyl butyrate	1	1	0.007
2-propyl-1-pentanol	1	1	0.002
1-Pentanol × 3-Octanone	1	1	0.743
1-Pentanol × Ethyl butyrate	1	1	0.329
1-Pentanol × 2-propyl-1-pentanol	1	1	0.576
3-Octanone × Ethyl butyrate	1	1	0.186
3-Octanone × 2-propyl-1-pentanol	1	1	0.031
Ethyl butyrate × 2-propyl-1-pentanol	1	1	0.054
1-Pentanol × 3-Octanone × Ethyl butyrate	1	1	0.676
1-Pentanol × 3-Octanone × 2-propyl-1-pentanol	1	1	0.658
1-Pentanol × Ethyl butyrate × 2-propyl-1-pentanol	1	1	0.167
3-Octanone × Ethyl butyrate × 2-propyl-1-pentanol	1	1	0.023
1-Pentanol × 3-Octanone × Ethyl butyrate × 2-propyl-1-pentanol	1		NA

## Data Availability

The data presented in this study are available within the article and its Supplementary Materials.

## Supplementary Materials

The following supporting information can be downloaded at: https://www.mdpi.com/article/10.3390/ani16101444/s1, Supplementary Table S1. Mushroom samples used for GC/MS analysis of volatile compounds; Supplementary Table S2-1. Volatile compounds detected from Amanita species. Imm., immature; Mat., mature; Sen., senescent. A plus sign (+) indicates that the compound was detected; Supplementary Table S2-2. Volatile compounds detected from other mushroom species. Imm., immature; Mat., mature; Sen., senescent. A plus sign (+) indicates that the compound was detected; Supplementary Table S3. Sample code used in the cluster analysis, showing fungal species, maturation stage, and sample form: immature; M, mature; O, senescent; P, fragment; W, intact; Supplementary Table S4. Volatile compounds detected from fruiting body fragments of *Amanita pallidorosea*. Presence/absence of each compound is indicated by 1 (detected) or 0 (not detected). Imm., immature; Mat., mature; Sen., senescent; Supplementary Table S5. Volatile compounds detected from whole fruiting bodies of *Amanita pallidorosea*. Presence/absence of each compound is indicated by 1 (detected) or 0 (not detected). Imm., immature; Mat., mature; Sen., senescent; Supplementary Table S6. Behavioral responses of three Drosophila species to candidate repellent compounds selected from fragmented fruiting bodies of *Amanita pallidorosea* (decay-stage odors). Counts on the paraffin side and odor-treated side were pooled across three trials for each treatment and compared using a chi-square test; Supplementary Table S7. Behavioral responses of three Drosophila species to candidate repellent compounds selected from intact fruiting bodies of *Amanita pallidorosea* (spore-dispersal stage). Counts on the odor-treated side were pooled across three trials for each treatment and compared using a chi-square test.

## Abbreviations

The following abbreviations are used in this manuscript:

DMTS	dimethyl trisulfide
VOCs	Volatile organic compounds
GC/MS	Gas Chromatography-Mass Spectrometry
1-Pe	1-Pentanol
PEA	Pheylethyl alcohol
